# Design, Synthesis and *in Vivo* Anti-inflammatory Activities of 2,4-Diaryl-5-4*H*-imidazolone Derivatives

**DOI:** 10.3390/molecules171012262

**Published:** 2012-10-18

**Authors:** Moustafa El-Araby, Abdelsattar Omar, Hassanein H. Hassanein, Abdel-Ghany H. El-Helby, Asharf A. Abdel-Rahman

**Affiliations:** 1 Pharmaceutical Organic Chemistry Department, Faculty of Pharmacy, Helwan University, Cairo 11790, Egypt; Email: alamka@yahoo.com; 2 Pharmaceutical Chemistry Department, Faculty of Pharmacy, King AbdulAziz University, Jeddah 21589, Saudi Arabia; 3 Pharmaceutical Chemistry Department, Faculty of Pharmacy, Al-Azhar University, Cairo 11884, Egypt; 4 Pharmaceutical Chemistry Department, Faculty of Pharmacy, Cairo University, Cairo 11787, Egypt

**Keywords:** anti-inflammatory, COX-2, COX-2 inhibitors, imidazolone, oxazolone, docking, structure-based design

## Abstract

A series of 2,4-diaryl-5(4*H*)-imidazolones were prepared and evaluated for their anti-inflammatory activities. Some selected 2,4-diaryl-5(4*H*)-imidazolones exhibited excellent anti-inflammatory activity in the carrageenan-induced rat paw edema model. Structure Activity Relationships within the series were studied. The substitution at the *N*-sulfonamide moiety by a small hydrophilic acetyl group resulted in compounds with superior *in vivo* anti-inflammatory properties. As expected from their COX-2 selectivity, most of the active compounds lacked gastrointestinal toxicity *in vivo* in rats after a 3-day treatment of 25 mg/kg/day.

## 1. Introduction

Cyclooxygenase-2 (COX-2) inhibition was identified two decades ago as the 21st century’s promising control for orthopedic pain and inflammation [1]. Celecoxib (Celebrex)™ was the first selective COX-2 inhibitor (coxibs) that appeared on the world markets in 1999 as a safer replacement for NSAIDs (non-selective COX-1/COX-2 inhibitors) as it causes less gastrointestinal complications [2]. After the launch of several successful anti-inflammatory medicines on world markets belonging to the selective COX-2 inhibitors class (Table 1), rofecoxib (Vioxx)™ and valdecoxib (Bextra)™ were withdrawn subsequent to evidence of atherothrombotic cardiovascular adverse effects (AEs) [3]. This dramatic turn raised serious questions about the safety of the COX-2 inhibition concept. Moreover, the U.S. FDA ordered explicit warnings on other marketed coxibs [4]. Fortunately, further studies revealed that cardiac adverse effects are related to certain drug structures and their metabolic byproducts rather than the COX-2 physiological role [5]. For instance, rofecoxib was hypothesized to produce highly reactive oxidized metabolites via oxidation of the core unsaturated lactone nucleus into a maleic anhydride peroxide radical species [6]. Rofecoxib may cause accumulation of oxidized LDL and 20-HETE, two biomarkers involved in atherosclerotic events [7,8]. Celecoxib was unable to produce similar metabolic products and hence, less cardiovascular associated risk was observed. Lumiracoxib is another selective COX-2 inhibitor that was also withdrawn due to non-cardiovascular toxicity issues [9,10].

**Table 1:** Selective inhibitors of COX-2. The structures of some examples of the first-generation (that is, rofecoxib and celecoxib) and second-generation (etoricoxib, lumiracoxib and valdecoxib). 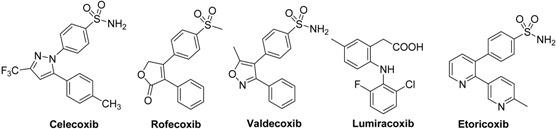

Approval	1999	1999	2001	2006	2005
Withdrawal	-------	2004	2005	2007	-------
Main AEs	-------	Cardiovascular	Cardiovascular	Hepatotoxicity	-------
Authority	FDA	FDA	FDA	EMA, TGA	UK

These findings led to a revitalization of medicinal chemistry research directed towards development of novel chemical classes of anti-inflammatory agents which are designed to act through inhibition of COX-2 [11,12,13]. Rapid progress in the discovery of novel anti-inflammatory agents may depend on their *in vivo* anti-inflammatory activities compared to ulcerogenic and other side effects [14]. For design purpose, it is useful to build on well established structural features of selective COX-2 inhibitors to maintain GI safety; and replace the central scaffold to avoid cardiovascular side effects. In this direction, we are presenting here compounds belonging to 1,2-diaryl-4-aylidene-5-4*H*-imidazolone with comparable anti-inflammatory potencies to reference NSAIDs, but with controlled ulcerogenic properties.

## 2. Results and Discussion

### 2.1. Rationale and Structure-Based Design

**Figure 1:**
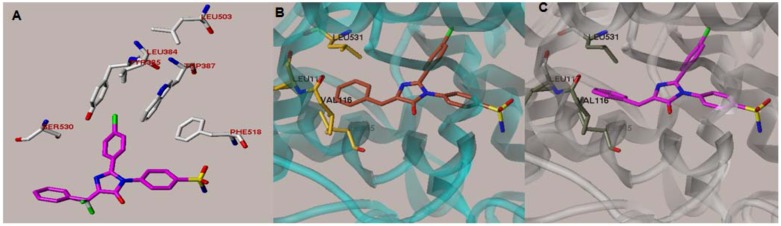
(**A**) Overlay of imidazolone **3a** and SC-558 inside the active site. Compound **3a** (magenta carbons) is completely eclipsing the structure of **SC-558** (white carbons). The figure also illustrates interaction of the C-2 *p*-fluorophenyl with hydrophobic amino acids. (**B**) Initial docking of compound **3a**,**b** (brown carbons) showing unfavorable approach of the arylidene group with Val-116. (**C**) Optimized docking after torsional changes in the arylidene moiety.

We envisaged 2,3-diaryl-5(4*H*)-imidazolones as promising molecular targets to develop novel COX-2 anti-inflammatory agents with selective COX-2 inhibition due to their similarity to celecoxib’s pyrazole core (Figure 1). The structure satisfies the basic requirements of selective COX-2 inhibitors as two adjacent aryl groups are attached to a heterocyclic core. One of the aryl groups is confined on the replacement of the methyl group of the lead celecoxib by fluorine, a small lipophilic group frequently employed as a metabolic blocker. A bulky lipophilic arylidene group on the imidazolone scaffold was used in place of celecoxib’s CF_3_ group. The side pocket, the major selectivity element towards COX-2 over COX-1, is occupied by the common phenylsulfonamide moeity. Some compounds contained an acetylsulfonamide, a water soluble group, to give a pharmacokinetic advantage. The acetylsulfonamido series was prepared as water soluble sodium salt.

**Scheme 1:**
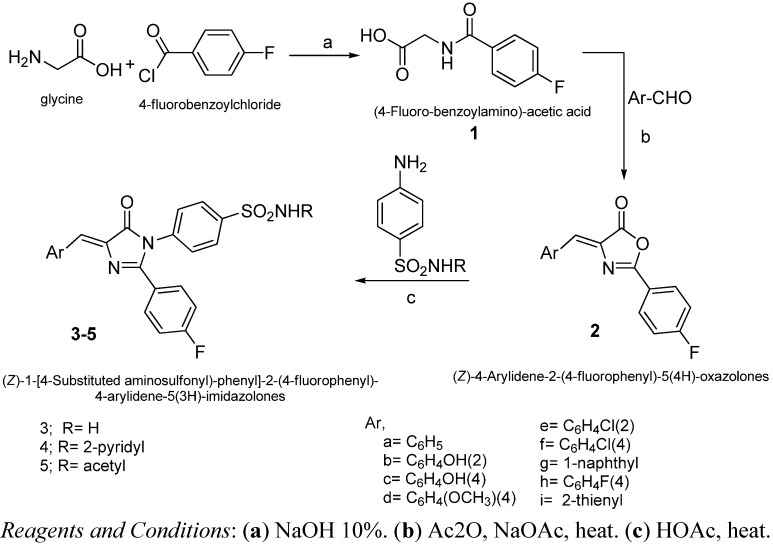
Synthesis of target compounds **3**–**5**.

The validity of our design was investigated using structure-based molecular modeling tools. The study was performed by docking the new imidazolones on the active site of the crystal structure of COX-2. All the molecular modeling works were performed on the SYBYL-X Suite [15]. Docking experiments were performed on the COX-2 structure coordinates downloaded from the Brookhaven Protein Databank (PDB entry: 6COX) [16]. This crystal structure complex contains the inhibitor SC-558, a closely related analogue of celecoxib. The ligand was extracted and modified to a representative of our compounds (**3a**, see Scheme 1 below). The geometry of the arylidene was adjusted to *Z* configuration because it was the least energy stereoisomer (Figure 1A).

The docking procedure revealed that fluorophenyl group maintains very good binding distances with the main hydrophobic channel residues Leu-384, Tyr-385, Trp-387, Leu-503, Phe-518 and the backbone of Ser-530.

**Figure 2:**
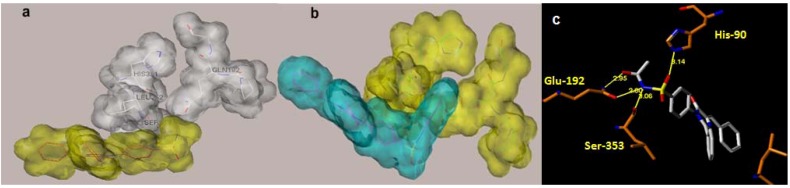
(**a**) The volume surface of the unsubstituted sulfonamide analogue (yellow) and the amino acid residues around. Note the narrow space available between Leu-352 and Gln-192. (**b**) *N*-pyridyl analogue (cyan) docked into the active site (yellow). Note the interference between the surface of the ligand and the protein residue. (**c**) Docking of *N*-acetyl derivative (white carbons) highlights favorable *H*-bond interactions of *N*-acetylsulfonamide group with residues of the active site.

The pocket around the sulfonamide is apparently hydrophilic as the sulfonamide group is interacting with the imidazole nitrogen of His-90 (one sulfonyl oxygen), the free amide group of glutamine at position 192 (the NH_2_ of the sulfonamide) and the NH of backbone nitrogen of Phe-518 (Figure 2a). The docking of *N*-pyridyl substituted sulfonamide showed a tight distance between the pyridyl ring and the backbone C=O of the Leu-352 causing the docking energy to become very high. Energy minimization of the new complex caused a slight shift in the peptide chain at this position with some distortion in bond angle of this carbonyl. Since this adjustment is not very likely in realty, this *N*-pyridyl series was expected to be less potent than the free sulfonamide group one. Volume surface illustration (Figure 2b) revealed that the pyridyl group would be possibly jammed in a narrow space that does not fit well in the active site. On the other hand, *N*-acetyl derivatives have reasonable size; hence, they fitted smoothly without need for computational optimizations. The added carbonyl established a new H-bond with the Gln-192 side chain, therefore, it synergizes the other hydrogen bond network of the sulfonamide NH forming a bifurcated H-bond with C=O of Gln-192 and Ser-353 (Figure 2c).

### 2.2. Chemistry

The compounds in the present work are prepared as described in Scheme 1. The key starting material 4-fluorobenzoylglycine (**1**) has been prepared by the reaction of 4-fluorobenzoyl chloride with glycine in aqueous sodium hydroxide solution. The hippuric acid derivative **1** underwent condensation with a set of aromatic aldehydes to obtain the oxazolone derivatives **2a**–**i** [17]. The target imidazolones **3**–**5** were prepared by the reaction of the oxazolones **2** with various substituted aromatic amines in boiling acetic acid [18]. The structures of all the newly synthesized compounds were elucidated with ^1^H-NMR, FT-IR and elemental analyses.

### 2.3. Biological Screening

#### 2.3.1. Anti-inflammatory Activity

The *in-vivo* anti-inflammatory activity was studied using carrageenan-induced rat paw edema test [19]. The anti-inflammatory activity of the tested compounds relative to that of indomethacin was also determined (Table 2).

**Table 2:** Effect of imidazolone compounds on carrageenan-induced rat paw edema (mL), % protection and activity relative to indomethacin and meloxicam.

Tested Compounds	Increase in paw edema (mL) ± SEM ^a,b^	% Protection	Activity relative to indomethacin
Control	0.96 ± 0.026	0.0	0.0
Indomethacin	0.25 ± 0.024	74.0	100
Meloxicam	0.23 ± 0.019	76.0	103
**3-a**	0.32 ± 0.028	66.7	90
**3-c**	0.42 ± 0.032	56.3	76
**3-d**	0.36 ± 0.016	62.5	84
**3-g**	0.31 ± 0.027	67.7	91
**3-h**	0.28 ± 0.022	70.8	96
**4-a**	0.64 ± 0.026	33.3	45
**4-c**	0.73 ± 0.022	24.0	32
**4-d**	0.71 ± 0.028	26.0	35
**4-g**	0.68 ± 0.032	29.2	39
**4-h**	0.49 ± 0.024	49.0	66
**5-a**	0.17 ± 0.016	82.3	111
**5-c**	0.27 ± 0.023	71.9	97
**5-d**	0.20 ± 0.024	79.2	107
**5-g**	0.19 ±0.029	80.2	108
**5-h**	0.08 ± 0.032	91.7	124

^a^ SEM denotes the standard error of the mean. ^b^ All data are significantly different from control (*p* < 0.001).

#### 2.3.2. Ulcerogenic Effects

Selected synthesized compounds were evaluated for their ulcerogenic potential in rats [20]. Indomethacin was used as reference standard. As shown in Table 3, most of tested compounds had much weaker ulcerogenic effect than indomethacin, but slightly higher than that of meloxicam.

**Table 3:** Anti-inflammatory (ED_50_, μM/kg) and ulcerogenic activity of imidazolones and reference drugs.

Compound	ED_50_ (μM/kg)	% Ulceration	Compound	ED_50_ (μM/kg)	% Ulceration
Indomethacin	9.7	100	**4-d**	29	20
Meloxicam	12	0	**4-g**	28	20
**3-a**	23	10	**4-h**	27	NT
**3-c**	25	NT	**5-a**	17.9	5
**3-d**	24	NT	**5-c**	18	10
**3-g**	22	NT	**5-d**	16.5	5
**3-h**	19.4	10	**5-g**	14.4	NT
**4-a**	28.7	25	**5-h**	12	5
**4-c**	31	NT			

NT = Not Tested.

### 2.4. Discussion

It is well established that scaffold-hopping can lead to new successful leads with better pharmacological profiles than older prototypes [21]. This approach was applied and lead to identification of compounds with good therapeutic index. The anti-inflammatory activity profile of our present compounds shows that the arylidene moiety replacing the CF_3_ of celecoxib had no detrimental effect on the anti-inflammatory activity. It was also proved that the *in silico* design work was beneficial in predicting activities of different compounds and hence, guiding the synthetic plan while reducing laboratory work as it leads to high *in vivo* activities. It also predicted lower activities of the *N*-pyridyl series. The outstanding potencies, low ulcerogenicity and excellent water solubility of *N*-acetylsulfonamide series **5** make it suitable for further development towards introduction of viable drug candidates. For instance, compound **5h** was as potent as the reference meloxicam with minimum ulcerogenic side effects on rats. In addition, this compound can be prepared and tested as a water soluble sodium salt to provide extra advantage in pharmacokinetic properties [22]. The model also revealed that the arylidene moiety may not cause steric conflicts with the active site although classic COX-2 inhibitors usually contain smaller groups at this position. Again, experimental results confirmed that the anti-inflammatory activity tolerates the presence of this group unless if it contains substitution on the *para* position. However, the anti-inflammatory results did not confirm the modeling projections regarding this relation. 

## 3. Experimental

### 3.1. General

All melting points are uncorrected and were determined by the open capillary tube method, using a Griffin melting point apparatus. IR spectra recorded on a Nicolet FT-IR-vector 22 instrument at the Pharmaceutical Analytical Unit, Faculty of Pharmacy, Al-Azhar University, Egypt, using KBr pellets and are expressed in cm^−1^. The ^1^H-NMR spectra were recorded on a Varian EM 390 (300 MHz) NMR spectrometer (in DMSO-d_6_) at the Microanalytical Center, Cairo University, Egypt, using TMS as internal reference and chemical shifts is measured in δ ppm. Mass spectra were recorded on a HP Model MS 5988 spectrometer at the Microanalytical Center, Cairo University, Egypt. Elemental analyses were performed using a Perkin Elmer 2400 Series II CHNS/O analyzer at the Pharmaceutical Analytical Unit, Faculty of Pharmacy, Al-Azhar University, Egypt. Progress of the reaction was monitored by TLC using sheets precoated with UV fluorescent silica gel Merck 60F 254.

*Preparation of 4-Fluorobenzoylglycine* (**1**) [23]. Glycine (0.03 mol, 2.5 g) was dissolved in sodium hydroxide solution (10%, 25 mL). 4-Fluorobenzoyl chloride (0.03 mol) was added portionwise with constant stirring for two hours. Crushed ice was added into the solution, and then it was slowly acidified with hydrochloric acid while stirring. The product was filtered, washed and crystallized from water; the mp 167–169 °C, lit [169–171 °C], yield 89%.

*General Procedure for Preparation of 4-Arylidene-2-(4-fluorophenyl)-5(4H)-oxazolones* (**2**) [18]. A mixture of 4-fluorobenzoylglycine (**1**, 0.01 mol, 2 g), the appropriate aromatic aldehyde (0.01 mol) and freshly fused sodium acetate (0.5 g) in acetic anhydride (20 mL) was heated at 100 °C for 2 h. The crystalline product obtained was filtered, washed with water then with aqueous ethanol and finally crystallized from ethanol.

*(Z)-4-Benzylidene-2-(4-fluorophenyl)-5(4H)-oxazolone* (**2a**) [24]. Yield: 72%; mp: 204–206 °C; ^1^H-NMR: 8.31–8.28 (m, 2H, CH aromatic) 8.22–8.17 (m, 2H, CH aromatic), 7.45–7.54 (m, 5H, CH aromatic), 7.39 (s, 1H, olefinic proton). IR: aromatic C–H stretch, 3073 cm^−1^; C=O stretch, 1794 cm^−1^; C=N stretch, 1652 cm^−1^; C=C stretch, 1597. MS *m/z* 267 (M^+^, 16.20%). Anal. Calcd for C_16_H_10_FNO_2_: C, 71.91; H, 3.77; N, 5.24. Found: C, 71.96; H, 3.73; N, 5.19.

*(Z)-2-(4-Fluorophenyl)-4-(2-hydroxybenzylidene)-5(4H)-oxazolone* (**2b**). Yield: 66%; mp: 194–195 °C; IR: O–H stretch, 3384 cm^−1^; aromatic C–H stretch, 3079 cm^−1^; C=O stretch, 1792 cm^−1^; C=N stretch, 1674 cm^−1^; C=C stretch, 1607. Anal. Calcd for C_16_H_10_FNO_3_: C, 67.84; H, 3.56; N, 4.94. Found: C, 67.78; H, 3.49; N, 4.98.

*(Z)-2-(4-Fluorophenyl)-4-(4-hydroxybenzylidene)-5(4H)-oxazolone* (**2c**). Yield: 85%; mp: 201–203 °C; ^1^H-NMR: 8.34–8.31 (d, 2H, *J* = 9 Hz, CH aromatic), 8.20–8.15 (*m*, 2H, CH aromatic), 7.49–7.43 (m, 2H, CH aromatic) 7.35 (s, 1H, olefinic proton), 7.31–7.27 (t, 2H, CH aromatic), 2.38 (s, 1H, OH proton). Anal. Calcd for C_16_H_10_FNO_3_: C, 67.84; H, 3.56; N, 4.94. Found: C, 67.76; H, 3.51; N, 4.97.

*(Z)-2-(4-Fluorophenyl)-4-(4-methoxybenzylidene)-5(4H)-oxazolone* (**2d**) [25]. Yield: 78%; mp: 189–191 °C; ^1^H-NMR: 8.30–8.18 (t, 2H, CH aromatic), 8.18–8.13 (d, 2H, CH aromatic), 7.49–7.43 (d, 2H, CH aromatic) 7.31 (s, 1H, olefinic proton), 7.11–7.08 (t, 2H, CH aromatic), 3.85 (s, 3H, CH_3_ proton). IR: aromatic C–H stretch, 3070 cm^−1^; C=O stretch, 1784 cm^−1^; C=N stretch, 1651 cm^−1^; C=C stretch, 1602. Anal. Calcd for C_17_H_12_FNO_3_: C, 68.68; H, 4.07; N, 4.71. Found: C, 68.71; H, 4.11; N, 4.67.

*(Z)-4-(2-Chlorobenzylidene)-2-(4-fluorophenyl)-5(4H)-oxazolone* (**2e**). Yield: 74%; mp: 220–223 °C; Anal. Calcd for C_16_H_9_ClFNO_2_: C, 63.70; H, 3.01; N, 4.64. Found: C, 63.65; H, 2.97; N, 4.58.

*(Z)-4-(4-Chlorobenzylidene)-2-(4-fluorophenyl)-5(4H)-oxazolone* (**2f**). Yield: 89%; mp: 228–230 °C; ^1^H-NMR: 8.25–8.12 (m, 4H, CH aromatic), 7.54–7.27 (m, 4H, CH aromatic), 7.25 (s, 1H, olefinic proton). IR: aromatic C–H stretch, 3057 cm^−1^; C=O stretch, 1792 cm^−1^; C=N stretch, 1656 cm^−1^; C=C stretch, 1592. Anal. Calcd for C_16_H_9_ClFNO_2_: C, 63.70; H, 3.01; N, 4.64. Found: C, 63.67; H, 2.95; N, 4.71.

*(Z)-2-(4-Fluorophenyl)-4-(1-naphthylidene)-5(4H)-oxazolone* (**2g**). Yield: 56%; mp: 217–219 °C; IR: aromatic C–H stretch, 3056 cm^−1^; C=O stretch, 1793 cm^−1^; C=N stretch, 1644 cm^−1^; C=C stretch, 1599. MS *m/z* 313 (M^+^, 6.3%). Anal. Calcd for C_20_H_12_FNO_2_: C, 75.70; H, 3.81; N, 4.41. Found: C, 75.63; H, 3.87; N, 4.56.

*(Z)-4-(4-Fluorobenzylidene)-2-(4-fluorophenyl)-5(4H)-oxazolone* (**2h**) [23]. Yield: 69%; mp: 214–216 °C; IR: aromatic C–H stretch, 3081 cm^−1^; C=O stretch, 1794 cm^−1^; C=N stretch, 1655 cm^−1^; C=C stretch, 1600. Anal. Calcd for C_16_H_9_F_2_NO_2_: C, 67.37; H, 3.18; N, 4.91. Found: C, 67.42; H, 3.25; N, 4.82.

*(Z)-2-(4-Fluorophenyl)-4-(2-thienylmethylene)-5(4H)-oxazolone* (**2i**) [26]. Yield: 78%; mp: 214–216 °C; IR: aromatic C–H stretch, 3081 cm^−1^; C=O stretch, 1794 cm^−1^; C=N stretch, 1655 cm^−1^; C=C stretch, 1600. Anal. Calcd for C_14_H_8_FNO_2_S: C, 61.53; H, 2.95; N, 5.13. Found: C, 61.56; H, 2.90; N, 5.17.

### 3.2. General Procedure for Preparation 3-[4-Substituted aminosulfonyl)-phenyl]-2-(4-fluorophenyl)-5-arylidene-5(4H)-imidazolones **3**–**5**

A mixture of the appropriate 4-arylidene-2-(4-fluorophenyl)-5(4*H*)-oxazolone **2** (0.01 mol) and 4-substituted aminobenzenesulfonamide (0.01 mol, 2.14 g) in glacial acetic acid (5 mL) containing freshly fused sodium acetate (0.3 g) was heated on boiling water bath with constant stirring for the appropriate time. The separated product was filtered, washed with aqueous ethanol and crystallized from ethanol.

*(Z)-1-[4-(Aminosulfonyl)-phenyl]-2-(4-fluorophenyl)-4-benzylidene-5(4H)-imidazolone* (**3a**). Prepared from **2a**. Yield: 62%; mp: 238–240 °C; IR: broad N–H stretch, 3450–3050 cm^−1^; overlap C=O stretch, amide band, 1603 cm^−1^; N–H bend, 1514 cm^−1^; asymmetric S(=O)_2_ stretch 1319 cm^−1^, symmetric S(=O)_2_ stretch 1161 cm^−1^. Anal. Calcd for C_22_H1_6_FN_3_O_3_S: C, 62.70; H, 3.83; N, 9.97. Found: C, 62.67; H, 3.79; N, 9.94.

*(Z)-1-[4-(Aminosulfonyl)-phenyl]-2-(4-fluorophenyl)-4-(2-hydroxybenzylidene)-5(4H)-imidazolone* (**3b**). Prepared from **2b**. Yield: 54%; mp: 267–269 °C; Anal. Calcd for C_22_H_16_FN_3_O_4_S: C, 60.40; H, 3.69; N, 9.61. Found: C, 60.37; H, 3.65; N, 9.63.

*(Z)-1-[4-(Aminosulfonyl)-phenyl]-2-(4-fluorophenyl)-4-(4-hydroxybenzylidene)-5(4H)-imidazolone* (**3c**). Prepared from **2c**. Yield: 64%; mp: 279–281 °C; ^1^H-NMR: 10.33 (s, 1H, OH), 10.03 (broad NH_2_ proton), 8.13–8.08 (m, 2H, CH aromatic), 7.98–7.86 (d, 2H, *J* = 9 Hz, CH aromatic), 7.78–7.75 (d, 2H, *J* = 9 Hz, CH aromatic), 7.51–7.48(d, 2H, *J* = 9 Hz, CH aromatic), 7.40–7.34 (m, 2H, CH aromatic), 7.15 (s, 1H, olefinic CH), 6.79–6.76 (d, 2H, *J* = 9 Hz, CH aromatic). Anal. Calcd for C_22_H_16_FN_3_O_4_S: C, 60.40; H, 3.69; N, 9.61. Found: C, 60.36; H, 3.72; N, 9.65.

*(Z)-1-[4-(Aminosulfonyl)-phenyl]-2-(4-fluorophenyl)-4-(4-methoxybenzylidene)-5(4H)-imidazolone* (**3d**). Prepared from **2d**. Yield: 76%; mp: 290–292 °C; IR: N–H stretch, 3260 cm^−1^; aromatic C–H stretch, 3105 cm^−1^; overlap C=O stretch, amide band, 1600 cm^−1^; N–H bend, 1514 cm^−1^; asymmetric S(=O)_2_ stretch 1330 cm^−1^, symmetric S(=O)_2_ stretch 1163 cm^−1^. ^1^H-NMR: 10.32 (broad NH_2_ proton), 8.14–8.09 (m, 2H, CH aromatic), 7.89–7.86 (d, 2H, *J* = 9 Hz, CH aromatic), 7.77–7.75 (d, 2H, *J* = 6 Hz, CH aromatic), 7.63–7.60 (d, 2H, *J* = 9 Hz, CH aromatic), 7.39–7.33 (m, 2H, CH aromatic), 7.17 (s, 1H, olefinic CH), 6.98–6.95 (d, 2H, *J* = 9 Hz, CH aromatic), 3.77 (s, 3H) OCH_3_. Anal. Calcd for C_23_H_18_FN_3_O_4_S: C, 61.19; H, 4.02; N, 9.31. Found: C, 61.15; H, 3.97; N, 9.28.

*(Z)-1-[4-(Aminosulfonyl)-phenyl]-4-(2-chlorobenzylidene)-2-(4-fluorophenyl)-5(4H)imidazolone* (**3e**). Prepared from **2e**. Yield: 79%; mp: 240–242 °C; Anal. Calcd for C_22_H_15_ClFN_3_O_3_: C, 57.96; H, 3.32; N, 9.22. Found: C, 57.91; H, 3.30; N, 9.26.

*(Z)-1-[4-(Aminosulfonyl)-phenyl]-4-(4-chlorobenzylidene)-2-(4-fluorophenyl)-5(4H)-imidazolone* (**3f**). Prepared from **2f**. Yield: 77%; mp: 239–241 °C; IR: broad N–H stretch, 3385–3117 cm^−1^; overlap C=O stretch, amide band, 1606 cm^−1^; N–H bend, 1482 cm^−1^; asymmetric S(=O)_2_ stretch 1324 cm^−1^, symmetric S(=O)_2_ stretch 1151 cm^−1^. Anal. Calcd for C_22_H_15_ClFN_3_O_3_: C, 57.96; H, 3.32; N, 9.22. Found: C, 57.99; H, 3.28; N, 9.19.

*(Z)-1-[4-(Aminosulfonyl)-phenyl]-2-(4-fluorophenyl)-4-(naphthalen-1-ylmethylene)-5(4H)-imidazolone* (**3g**). Prepared from **2g**. Yield: 67%; mp: 218–220 °C; Anal. Calcd for C_26_H_18_FN_3_O_3_S: C, 66.23; H, 3.85; N, 8.91. Found: C, 66.17; H, 3.92; N, 8.82.

*(Z)-1-[4-(Aminosulfonyl)-phenyl]-4-(4-fluorobenzylidene)-2-(4-fluorophenyl)-5(4H)-imidazolone* (**3h**). Prepared from **2h**. Yield: 72%; mp: 270–272 °C; Anal. Calcd for C_22_H_15_F_2_N_3_O_3_S: C, 60.13; H, 3.44; N, 9.56. Found: C, 60.16; H, 3.47; N, 9.60.

*(Z)-1-[4-(Aminosulfonyl)-phenyl]-2-(4-fluorophenyl)-4-(thien-2-ylmethylene)-5(4H)-imidazolone* (**3i**). Prepared from **2i**. Yield: 72%; mp: 245–247 °C; Anal. Calcd for C_20_H_14_FN_3_O_3_S_2_: C, 56.19; H, 3.30; N, 9.83. Found: C, 56.21; H, 3.26; N, 9.87.

*(Z)-4-Benzylidene-2-(4-fluorophenyl)-1-[4-(2-pyridylaminosulfonyl)-phenyl]-5(4H)-imidazolone* (**4a**). Prepared from **2a**. Yield: 73%; mp: 255–257 °C; Anal. Calcd for C_27_H_19_FN_4_O_3_S: C, 65.05; H, 3.84; N, 11.24. Found: C, 65.11; H, 3.87; N, 11.19.

*(Z)-2-(4-Fluorophenyl)-4-(2-hydroxybenzylidene)-1-[4-(2-pyridylaminosulfonyl)phenyl]-5(4H)-imidazolone* (**4b**). Prepared from **2b**. Yield: 43%; mp: 273–275 °C; Anal. Calcd for C_27_H_19_FN_4_O_4_S: C, 63.03; H, 3.72; N, 10.89. Found: C, 63.07; H, 3.69; N, 10.87.

*(Z)-2-(4-Fluorophenyl)-4-(4-hydroxybenzylidene)-1-[4-(2-pyridylaminosulfonyl)phenyl]-5(4H)-imidazolone* (**4c**). Prepared from **2c**. Yield: 72%; mp: 294–296 °C; IR: broad N–H stretch, 3410–3085 cm^−1^; overlap C=O stretch, amide band, 1604 cm^−1^; N–H bend, 1515 cm^−1^; asymmetric S(=O)_2_ stretch 1385 cm^−1^, symmetric S(=O)_2_ stretch 1147 cm^−1^. ^1^H-NMR: 10.47 (s, 1H, OH), 10.01 (s, 1H, NH), 8.11–8.00 (m, 2H, CH aromatic), 7.85–7.78 (m, 4H, CH aromatic), 7.69–7.63 (m, 2H, CH aromatic), 7.50–7.47 (m, 2H, CH aromatic), 7.38–7.32 (m, 2H, CH aromatic), 7.18–7.08 (m, 3H, 2CH aromatic, 1CH olefinic), 6.85–6.75 (m, 2H, CH aromatic). Anal. Calcd for C_27_H_19_FN_4_O_4_S: C, 63.03; H, 3.72; N, 10.89. Found: C, 63.08; H, 3.75; N, 10.92.

*(Z)-2-(4-Fluorophenyl)-4-(4-methoxybenzylidene)-1-[4-(2-pyridylaminosulfonyl)phenyl]-5(4H)-imidazolone* (**4d**). Prepared from **2d**. Yield: 72%; mp: 293–295 °C; IR: N–H stretch, 3290 cm^−1^; aromatic C–H stretch, 3060 cm^−1^; overlap C=O stretch, amide band, 1604 cm^−1^; N–H bend, 1510 cm^−1^; asymmetric S(=O)_2_ stretch 1383 cm^−1^, symmetric S(=O)_2_ stretch 1153 cm^−1^. ^1^H-NMR: 10.38 (s, 1H, NH), 8.11–8.07 (m, 2H, CH aromatic), 7.83–7.82 (d, 2H, *J* = 3 Hz, CH aromatic), 7.67–7.57 (m, 4H, CH aromatic), 7.39–7.33 (m, 2H, CH aromatic), 7.14–7.09 (m, 5H, 4CH aromatic, 1CH olefinic), 6.97–6.94 (d, 2H, *J* = 9 Hz, CH aromatic), 3.76 (s, 3H) OCH_3_. MS *m/z* 528 (M^+^, 50.10%). Anal. Calcd for C_28_H_21_FN_4_O_4_S: C, 63.63; H, 4.00; N, 10.60. Found: C, 63.57; H, 4.03; N, 10.67.

*(Z)-4-(2-Chlorobenzylidene)-2-(4-fluorophenyl)-1-[4-(2-pyridylaminosulfonyl)phenyl]-5(4H)-imidazolone* (**4e**). Prepared from **2e**. Yield: 84%; mp: 244–246 °C; Anal. Calcd for C_27_H_18_ClFN_4_O_3_S: C, 60.85; H, 3.40; N, 10.51. Found: C, 60.87; H, 3.37; N, 10.46.

*(Z)-4-(4-Chlorobenzylidene)-2-(4-fluorophenyl)-1-[4-(2-pyridylaminosulfonyl)phenyl]-5(4H)-imidazolone* (**4f**). Prepared from **2f**. Yield: 89%; mp: 245–247 °C; ^1^H-NMR: 10.17 (s, 1H, NH), 8.09–8.01 (m, 2H, CH aromatic), 7.84 (m, 2H, CH aromatic), 7.64–7.61 (m, 4H, CH aromatic), 7.48–7.45 (d, 2H, *J* = 9 Hz, CH aromatic), 7.38–7.33 (m, 2H, CH aromatic), 7.14–7.09 (m, 3H, 2CH aromatic, 1CH olefinic), 6.87 (m, 2H, CH aromatic). MS *m/z* 531 (M^+^, 13.50%). Anal. Calcd for C_27_H_18_ClFN_4_O_3_S: C, 60.85; H, 3.40; N, 10.51. Found: C, 60.87; H, 3.43; N, 10.47.

*(Z)-2-(4-Fluorophenyl)-4-(naphthalen-1-ylmethylene)-1-[4-(2-pyridylaminosulfonyl)phenyl]-5(4H)-imidazolone* (**4g**). Prepared from **2g**. Yield: 76%; mp: 236–238 °C; IR: N–H stretch, 3303 cm^−1^; aromatic C–H stretch, 3061 cm^−1^; overlap C=O stretch, amide band, 1648 cm^−1^; N–H bend, 1496 cm^−1^; asymmetric S(=O)_2_ stretch 1338 cm^−1^. MS *m/z* 548 (M^+^, 21.10%). Anal. Calcd for C_31_H_21_FN_4_O_3_S: C, 67.87; H, 3.86; N, 10.21. Found: C, 67.82; H, 3.91; N, 10.15.

*(Z)-4-(4-Fluorobenzylidene)-2-(4-fluorophenyl)-1-[4-(2-pyridylaminosulfonyl)phenyl]-5(4H)-imidazolone* (**4h**). Prepared from **2h**. Yield: 81%; mp: 250–252 °C; Anal. Calcd for C_27_H_18_F_2_N_4_O_3_S: C, 62.78; H, 3.51; N, 10.85. Found: C, 62.81; H, 3.54; N, 10.82.

*(Z)-2-(4-Fluorophenyl)-1-[4-(2-pyridylaminosulfonyl)phenyl]-4-(thien-2-ylmethylene)-5(4H)-imidazolone* (**4i**). Prepared from **2i**. Yield: 76%; mp: 251–252 °C; Anal. Calcd for C_25_H_17_FN_4_O_3_S_2_: C, 59.51; H, 3.40; N, 11.10. Found: C, 59.54; H, 3.36; N, 11.14.

*(Z)-1-[4-(Acetylaminosulfonyl)phenyl]-4-benzylidene-2-(4-fluorophenyl)-5(4H)-imidazolone* (**5a**). Prepared from **2a**. Yield: 43%; mp: 231–233 °C; ^1^H-NMR: 10.53 (s, 1H, NH), 8.10–8.08 (m, 2H, CH aromatic), 7.92–7.85 (m, 2H, CH aromatic), 7.69–7.63 (m, 2H, CH aromatic), 7.38–7.33 (m, 5H, CH aromatic), 7.31–7.30 (d, 2H, *J* = 3 Hz, CH aromatic), 7.19 (s, 1H, CH olefinic), 2.5 (s, 3H) CH_3_. Anal. Calcd for C_24_H_18_FN_3_O_4_S: C, 62.19; H, 3.91; N, 9.07. Found: C, 62.22; H, 3.87; N, 9.11.

*(Z)-1-[4-(Acetylaminosulfonyl)-phenyl]-2-(4-fluorophenyl)-4-(2-hydroxybenzylidene)-5(4H)-imidazolone* (**5b**). Prepared from **2b**. Yield: 38%; mp: 255–257 °C; Anal. Calcd for C_24_H_18_FN_3_O_5_S: C, 60.12; H, 3.78; N, 8.76. Found: C, 60.07; H, 3.81; N, 8.80.

*(Z)-1-[4-(Acetylaminosulfonyl)-phenyl]-2-(4-fluorophenyl)-4-(4-hydroxybenzylidene)-5(4H)-imidazolone* (**5c**). Prepared from **2c**. Yield: 48%; mp: 279–281 °C; Anal. Calcd for C_24_H_18_FN_3_O_5_S: C, 60.12; H, 3.78; N, 8.76. Found: C, 60.15; H, 3.82; N, 8.81.

*(Z)-1-[4-(Acetylaminosulfonyl)-phenyl]-2-(4-fluorophenyl)-4-(4-methoxybenzylidene)-5(4H)-imidazolone* (**5d**). Prepared from **2d**. Yield: 53%; mp: 285–287 °C; IR: N–H stretch, 3240 cm^−1^; aromatic C–H stretch, 3110 cm^−1^; overlap C=O stretch, amide band, 1650 cm^−1^; N–H bend, 1500 cm^−1^; asymmetric S(=O)_2_ stretch 1340 cm^−1^, symmetric S(=O)_2_ stretch 1162 cm^−1^. Anal. Calcd for C_25_H_20_FN_3_O_5_S: C, 60.84; H, 4.08; N, 8.51. Found: C, 60.87; H, 4.11; N, 8.56.

*(Z)-1-[4-(Acetylaminosulfonyl)-phenyl]-4-(2-chlorobenzylidene)-2-(4-fluorophenyl)-5(4H)-imidazolone* (**5e**). Prepared from **2e**. Yield: 48%; mp: 241–243 °C; Anal. Calcd for C_24_H_17_ClFN_3_O_4_S: C, 57.89; H, 3.44; N, 8.44. Found: C, 57.86; H, 3.39; N, 8.47.

*(Z)-1-[4-(Acetylaminosulfonyl)-phenyl]-4-(4-chlorobenzylidene)-2-(4-fluorophenyl)-5(4H)-imidazolone* (**5f**). Prepared from **2f**. Yield: 64%; mp: 237–239 °C; Anal. Calcd for C_24_H_17_ClFN_3_O_4_S: C, 57.89; H, 3.44; N, 8.44. Found: C, 57.88; H, 3.47; N, 8.42.

*(Z)-1-[4-(Acetylaminosulfonyl)-phenyl]-2-(4-fluorophenyl)-4-(naphthalen-1-ylmethylene)-5(4H)-imidazolone* (**5g**). Prepared from **2g**. Yield: 51%; mp: 231–233 °C; Anal. Calcd for C_28_H_20_FN_3_O_4_S: C, 65.49; H, 3.93; N, 8.18. Found: C, 65.53; H, 3.95; N, 8.25.

*(Z)-1-[4-(Acetylaminosulfonyl)-phenyl]-4-(2-fluorobenzylidene)-2-(4-fluorophenyl)-5(4H)-imidazolone* (**5h**). Prepared from **2h**. Yield: 54%; mp: 250–253 °C; Anal. Calcd for C_24_H_17_F_2_N_3_O_4_S: C, 59.87; H, 3.56; N, 8.73. Found: C, 59.92; H, 3.61; N, 8.77.

*(Z)-1-[4-(Acetylaminosulfonyl)-phenyl]-2-(4-fluorophenyl)-4-(thien-2-ylmethylene)-5(4H)-imidazolone* (**5i**). Prepared from **2i** and recrystallized from ethanol. Yield: 43%; mp: 237–239 °C; Anal. Calcd for C_22_H_16_FN_3_O_4_S_2_: C, 56.28; H, 3.43; N, 8.95. Found: C, 56.31; H, 3.40; N, 8.97.

### 3.3. Anti-inflammatory Test

Male albino rats weighing 150–180 g (National Research Institute, Cairo) were used throughout the work. They were kept in the animal house under standard conditions of light and temperature with free access to food and water. The animals were randomly divided into groups of six rats each. The paw edema was induced by sub-plantar injection of 50 µL of 1% carrageenan solution in saline (0.9%). Indomethacin, meloxicam and the test compounds were dissolved in DMSO and injected subcutaneously in different dose levels of 1, 5 and 10 mg/kg body weight respectively, 1 h prior to carrageenan injection. DMSO was injected to the control group. The volume of paw edema (in mL) was determined by means of a water plethysmometer immediately after injection of carrageenan and 4 h later. ED_50_ was calculated for the test compounds and reference drugs through dose response curves by measuring the inhibition of edema volume 4 h after the carrageenan injection. The percentage protection against inflammation was calculated as follows: Vc − Vd/Vc × 100, where Vc is the increase in paw volume in the absence of the test compound (control) and Vd is the increase of paw volume after injection of the test compound. Data were expressed as means ± SEM. Significant differences between the control and the treated groups were obtained using Student’s t-test and *p*-values. The differences in results were considered significant when *p* < 0.001.

### 3.4. Ulcerogenicity Test

Male albino rats (120–150 g) were fasted for 12 h prior to the administration of the compounds. The animals were divided into groups, each of six animals. The control group received 0.2 mL DMSO orally, reference groups received 5 mg/kg indomethacin and test groups received 10 mg/kg tested compounds orally for three successive days. Animals were sacrificed by diethyl ether 6 h after the last dose and the stomach was removed. An opening at the greater curvature was made and the stomach was cleaned by washing with cold saline and inspected with a 3× magnifying lens for any evidence of hyperemia, hemorrhage, definite hemorrhagic erosion, or ulcer. An arbitrary scale was used to calculate the ulcer index which indicates the severity of the stomach lesions (Table 3). The % ulceration for each group was calculated as follows:

% Ulceration = Number of animals bearing ulcer in a group/Total number of animals in the same group × 100.

## 4. Conclusions

The arylidene-5-4*H*-imidazolone framework was proved to be a promising molecular target to develop anti-inflammatory agents. COX-2 inhibition, though not tested, is the likely mechanism of action as predicted by molecular docking experiments. Water soluble derivatives of the present scaffold were effective *in vivo* compared to reference marketed drugs, providing an extra molecular druggability feature. The present imidazolone anti-inflammatory compounds showed weak ulcerogenic side effects which are comparable, but not superior, to the positive reference. Further biochemical and pharmacological studies are undergoing to optimize their pharmacological profile and to explore the exact mechanism of action.
